# Supplementary material for: When less is more powerful: Shapley value attributed ablation with augmented learning for practical time series sensor data classification
Source: PLoS One. 2022 Nov 23;17(11):e0277975. doi: 10.1371/journal.pone.0277975 (PMC9683574; doi:10.1371/journal.pone.0277975)
Supplement: S1 Table — The hyperparameters used in ShapAAL model construction. (PDF) [file pone.0277975.s002.pdf]

Hyperparameter	value or attribute
Number of iterations (epochs)	1000
Learning rate	10^{-3}
Loss function	Categorical cross-entropy
Optimizer	Adam
Kernel size (for seven convolutional layers)	15, 13, 11, 9, 7, 5, 3
Stride length	1
Padding	Same (zero padding)
