# Supplementary figures and images for: When less is more powerful: Shapley value attributed ablation with augmented learning for practical time series sensor data classification
Source: PLoS One. 2022 Nov 23;17(11):e0277975. doi: 10.1371/journal.pone.0277975 (PMC9683574; doi:10.1371/journal.pone.0277975)

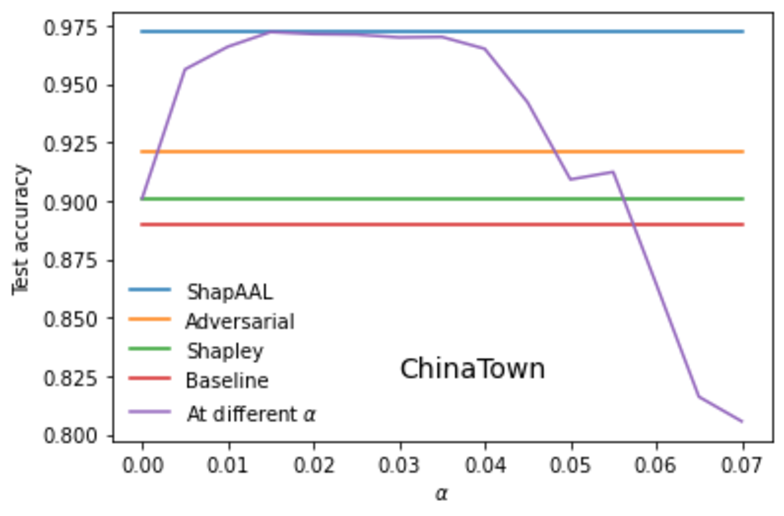

Supplement: S1 Fig — We depict the trend of the test accuracy data augmentation control parameter α in ChinaTown dataset by varying α from 0.00 ≤ α ≤ 0.07 to understand the response of the model under different strengths of perturbations. (TIF) [file pone.0277975.s003.tif]

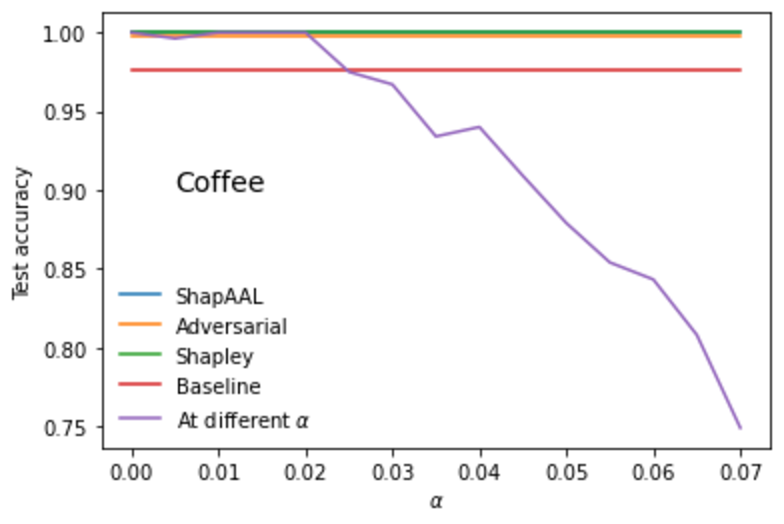

Supplement: S2 Fig — We depict the trend of the test accuracy data augmentation control parameter α in Coffee dataset by varying α from 0.00 ≤ α ≤ 0.07 to understand the response of the model under different strengths of perturbations. (TIF) [file pone.0277975.s004.tif]

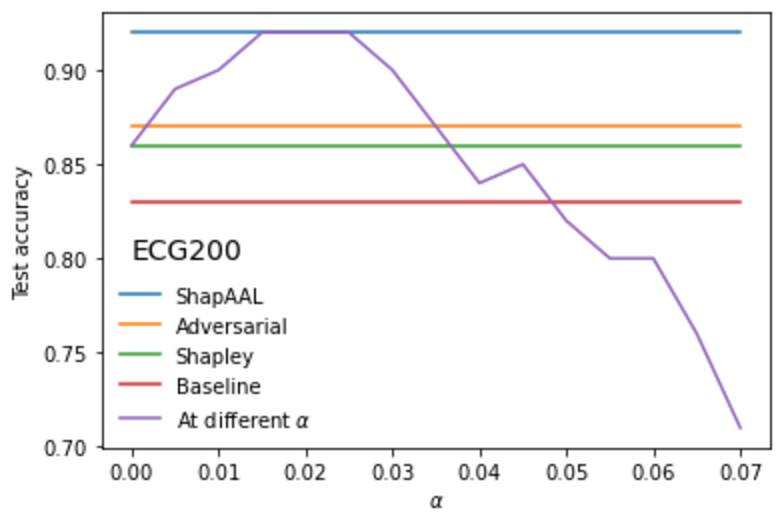

Supplement: S3 Fig — We depict the trend of the test accuracy data augmentation control parameter α in ECG200 dataset by varying α from 0.00 ≤ α ≤ 0.07 to understand the response of the model under different strengths of perturbations. (TIF) [file pone.0277975.s005.tif]

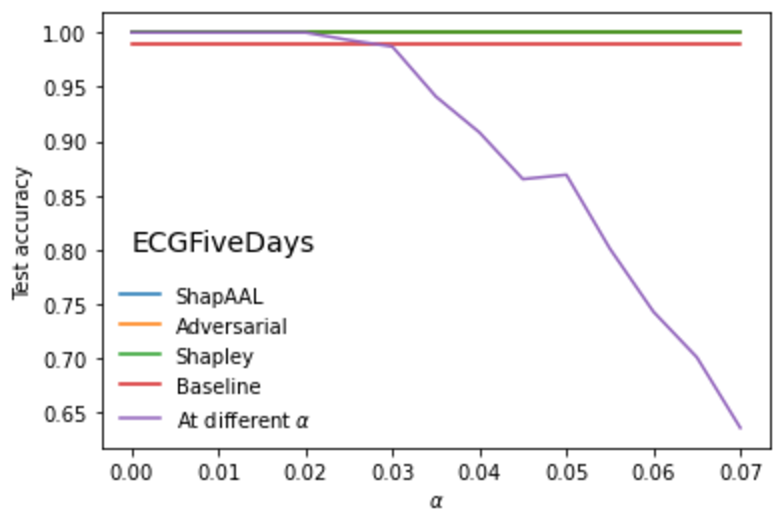

Supplement: S4 Fig — We depict the trend of the test accuracy data augmentation control parameter α in ECGFiveDays dataset by varying α from 0.00 ≤ α ≤ 0.07 to understand the response of the model under different strengths of perturbations. (TIF) [file pone.0277975.s006.tif]

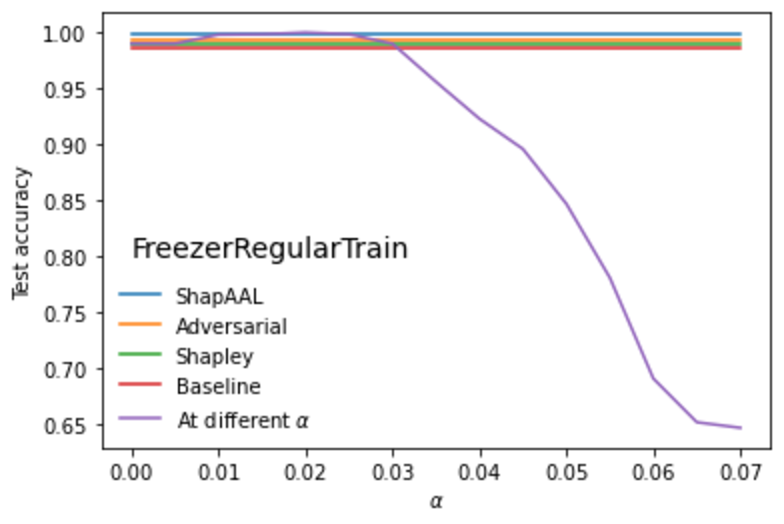

Supplement: S5 Fig — We depict the trend of the test accuracy data augmentation control parameter α in FreezerRegularTrain dataset by varying α from 0.00 ≤ α ≤ 0.07 to understand the response of the model under different strengths of perturbations. (TIF) [file pone.0277975.s007.tif]

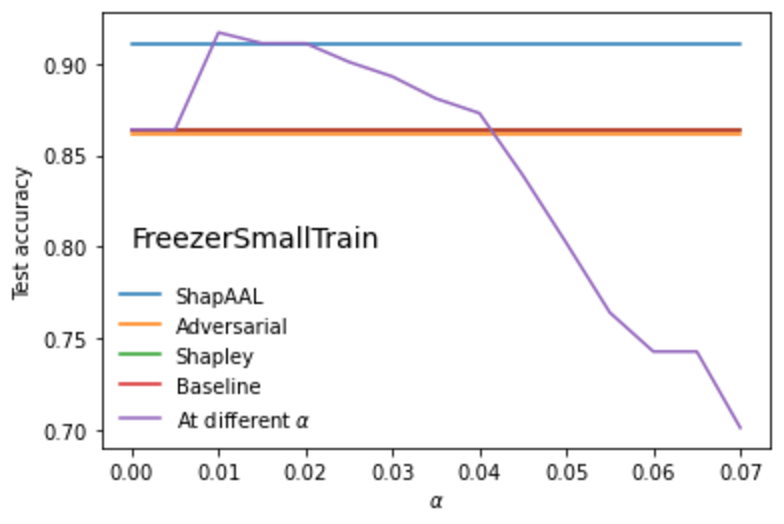

Supplement: S6 Fig — We depict the trend of the test accuracy data augmentation control parameter α in FreezerSmallTrain dataset by varying α from 0.00 ≤ α ≤ 0.07 to understand the response of the model under different strengths of perturbations. (TIF) [file pone.0277975.s008.tif]

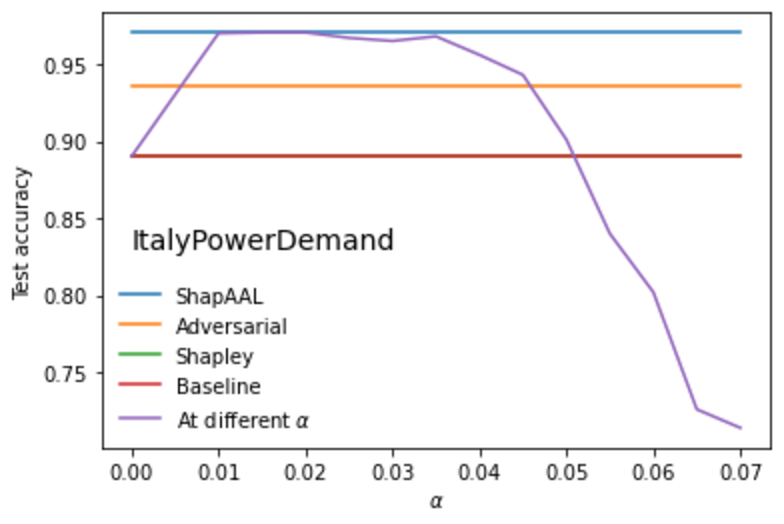

Supplement: S7 Fig — We depict the trend of the test accuracy data augmentation control parameter α in ItalyPowerDemandn dataset by varying α from 0.00 ≤ α ≤ 0.07 to understand the response of the model under different strengths of perturbations. (TIF) [file pone.0277975.s009.tif]

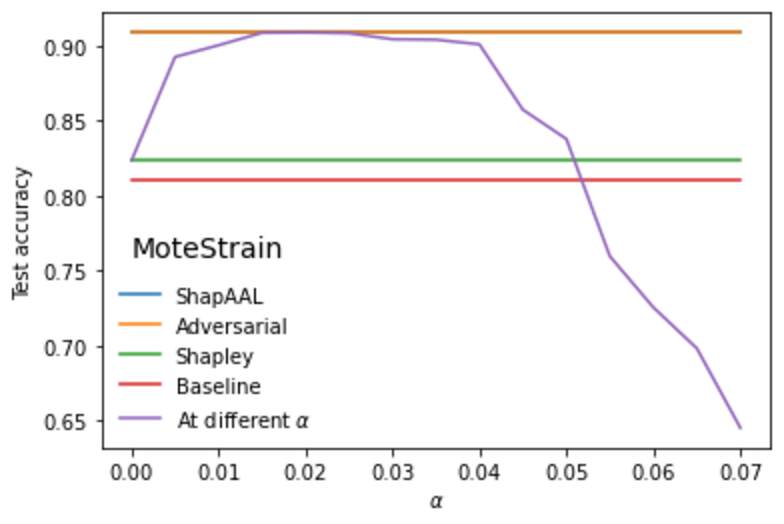

Supplement: S8 Fig — We depict the trend of the test accuracy data augmentation control parameter α in MoteStrain dataset by varying α from 0.00 ≤ α ≤ 0.07 to understand the response of the model under different strengths of perturbations. (TIF) [file pone.0277975.s010.tif]

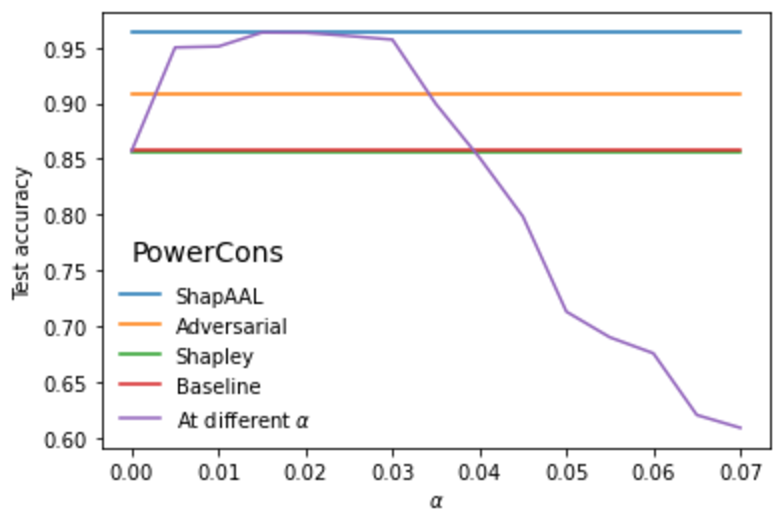

Supplement: S9 Fig — We depict the trend of the test accuracy data augmentation control parameter α in PowerCons dataset by varying α from 0.00 ≤ α ≤ 0.07 to understand the response of the model under different strengths of perturbations. (TIF) [file pone.0277975.s011.tif]

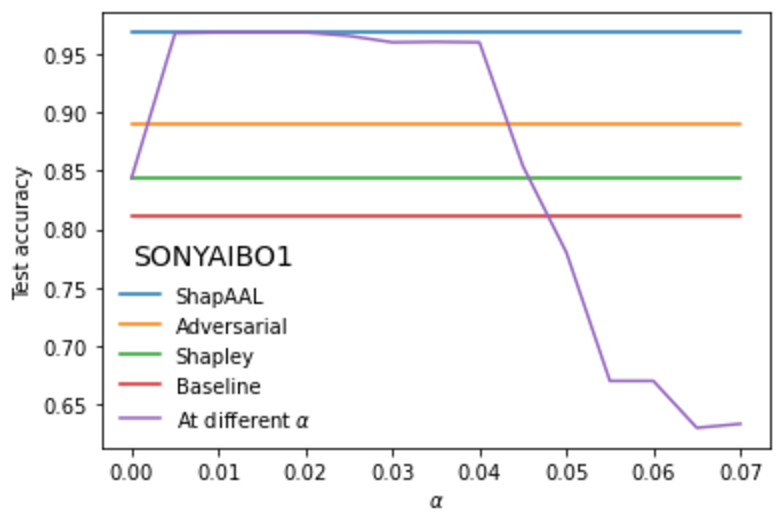

Supplement: S10 Fig — We depict the trend of the test accuracy data augmentation control parameter α in SonyAIBO1 dataset by varying α from 0.00 ≤ α ≤ 0.07 to understand the response of the model under different strengths of perturbations. (TIF) [file pone.0277975.s012.tif]

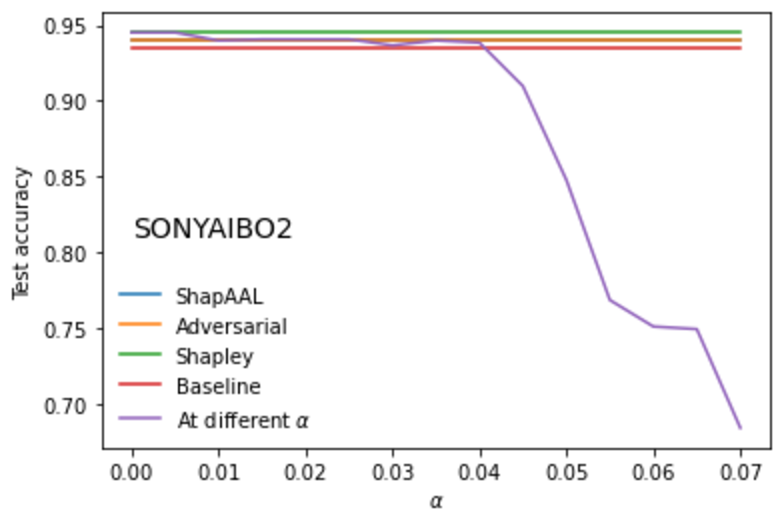

Supplement: S11 Fig — We depict the trend of the test accuracy data augmentation control parameter α in SonyAIBO2 dataset by varying α from 0.00 ≤ α ≤ 0.07 to understand the response of the model under different strengths of perturbations. (TIF) [file pone.0277975.s013.tif]

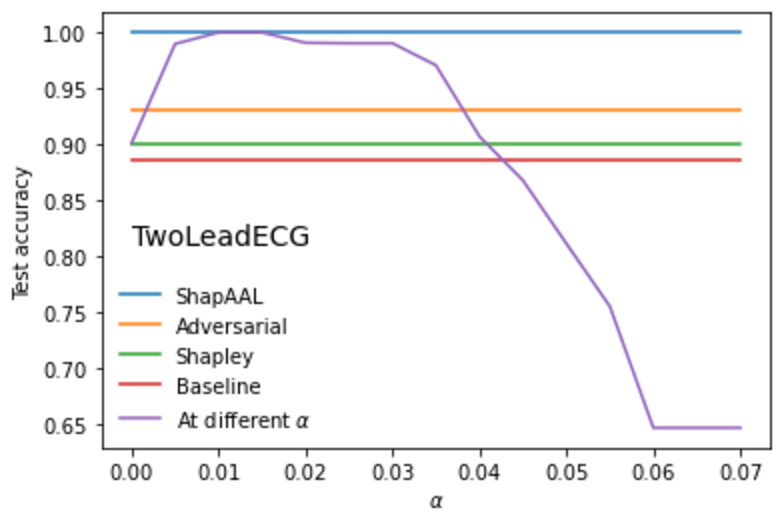

Supplement: S12 Fig — We depict the trend of the test accuracy data augmentation control parameter α in TwoLeadECG dataset by varying α from 0.00 ≤ α ≤ 0.07 to understand the response of the model under different strengths of perturbations. (TIF) [file pone.0277975.s014.tif]

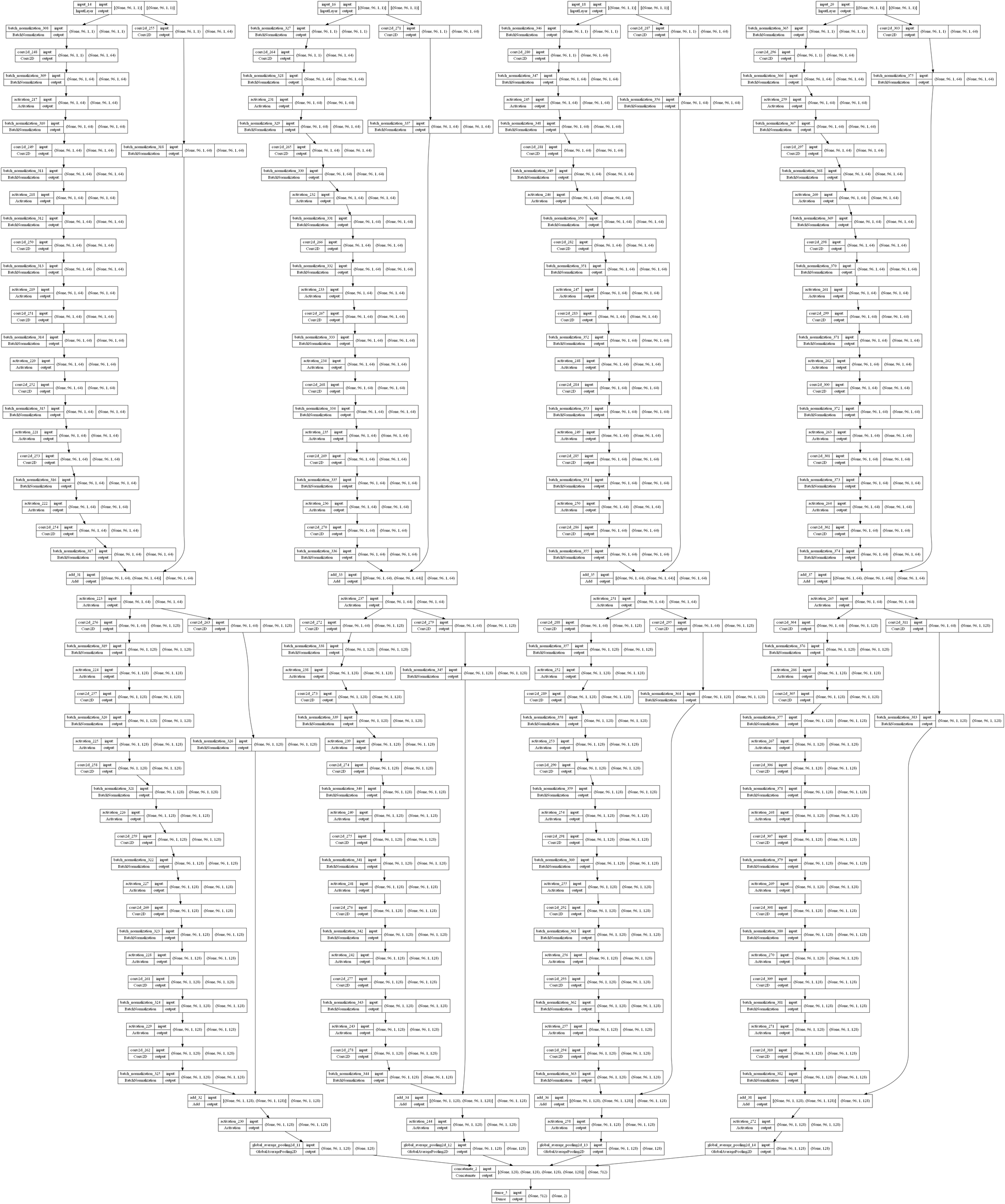

Supplement: S13 Fig — We present the complete model description for reproducibility, where the input is “ECG200” training dataset. (TIF) [file pone.0277975.s015.tif]
